# The Complicated Art of Tracking Dengue

**DOI:** 10.1371/journal.pmed.0020116

**Published:** 2005-04-26

Compared with malaria, dengue fever has a rather lower profile in the public mind, although to those who have had it, it leaves a great impression. The name dengue fever is derived from the Swahiliwords *Ki denga pepo* (“it is a sudden overtaking by an evil spirit”), which gives an idea of the rapid onset of the disease. The dengue virus is carried by the mosquito Aedes aegypti, and the disease often occurs as epidemics. Although the classic illness is a fairly benign acute febrile syndrome, it may be very painful—hence the English nickname, breakbone fever. The virus can also cause a much more serious illness known as dengue hemorrhagic fever, which can progress to dengue shock syndrome. There are four main serotypes of the dengue RNA virus; dengue hemorrhagic fever is more likely to occur during dengue infection in people with preexisting active or passive (e.g., maternally acquired) immunity who are exposed to a different dengue virus serotype. In contrast to classic dengue, the hemorrhagic fever and shock syndromes are mostly diseases of children and, if untreated, have a mortality of around 50%.[Fig pmed-0020116-g001]

**Figure pmed-0020116-g001:**
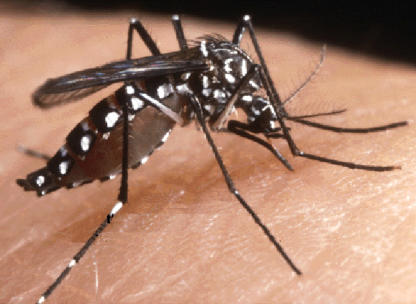
Aedes aegypti, the main vector of dengue (Photo: CDC/Robert S Craig)

Around two-fifths of the world's population are now at risk of the disease (one estimate is that 80 million people are infected each year). The number at risk will increase as population growth, urbanization, international travel, and climate change influence transmission of the disease. Understanding how all these factors interact is important in planning for disease outbreaks. However, the incidence of dengue is not easily predictable, varying with season, and also between years. For example, although dengue is most prevalent in the wet season, dengue epidemics have also been associated with drought in some countries. El Niño is the best known climatic event affecting climate between years, and some research already suggests that there is a relationship between the timing of dengue epidemics and El Niño in the Pacific Islands and in other countries.

Previous research has uncovered traveling waves of dengue in Thailand, but the cause of these has been obscure. In a paper in this month's *PLoS Medicine* Bernard Cazelles and colleagues looked at the details of the relationship between dengue incidence and El Niño in Thailand. Their results, based on complex mathematical analysis, do not provide easy answers for those who might want to plan for dengue outbreaks, though they do go some way to helping to understand the complex interplay between the various factors. In essence, the researchers found that there was a significant association between El Niño oscillations, climate variables, and dengue hemorrhagic fever incidence with a 2- to 3-year repeat, for both Bangkok and the rest of Thailand. However this association was significant only for the years 1986–1992, and outside these years factors other than climate were probably responsible for triggering the disease outbreaks.

